# Huatuo Zaizao pill promotes functional recovery and neurogenesis after cerebral ischemia-reperfusion in rats

**DOI:** 10.1186/s12906-016-1516-z

**Published:** 2017-01-05

**Authors:** Sijin Duan, Tian Wang, Jianqiao Zhang, Minmin Li, Chengwen Lu, Lijie Wang, Yan Zou, Fenghua Fu

**Affiliations:** 1 School of Pharmacy, Key Laboratory of Molecular Pharmacology and Drug Evaluation, Yantai University, Yantai, 264005 People’s Republic of China; 2 Ministry of Education, Collaborative Innovation Center of Advanced Drug Delivery System and Biotech Drugs in Universities of Shandong, Yantai University, Yantai, 264005 People’s Republic of China

**Keywords:** Huatuo Zaizao pill, Stroke, Functional recovery, Cerebral ischemia-reperfusion, Neurogenesis

## Abstract

**Background:**

Ischemic stroke is the third leading cause of death in adults worldwide and is the first leading cause of long-term disability. Neurogenesis plays an important role in promoting behavioral recovery after stroke. Huatuo Zaizao pill (HT), a traditional Chinese medicine, has been used clinically in China to promote the rehabilitation after stroke, but the underlying mechanism of action was still unclear. This study is to investigate the effects of HT on the functional recovery in a rat model of cerebral ischemia-reperfusion (I/R) injury, and the potential molecular mechanisms.

**Methods:**

Rats were randomly divided into sham, model with cerebral I/R injury, or HT-treated groups, then administered orally with vehicle (for the sham and model group) or HT (0.5, 1.0, or 2.0 mg/kg) respectively, for 3 or 7 days. Functional recovery was assessed by cylinder test, beam walking test, and adhesive test. Neurogenesis was investigated by double immunofluorescence staining for 5-ethynyl-2-deoxyuridine (EdU) and neuronal nuclear protein (NeuN). The proteins of kinase A (PKA), cAMP response element-binding protein (CREB), and brain-derived neurotrophic factor (BDNF) were assayed by western blotting. The level of BDNF mRNA was evaluated by RT-PCR.

**Results:**

Compared with the model group, treatment with HT significantly promoted functional recovery in I/R injured rats (*p* < 0.05 or *p* < 0.01). The generation of new neurons was increased in the HT groups. HT treatment for 3 days increased the level of BDNF mRNA in I/R injured rats. Expression of PKA, phosphorylated CREB, and BDNF were significantly (*p* < 0.05) increased with the 7-day HT treatment.

**Conclusions:**

These results indicated that HT treatment could promote functional recovery after stroke. HT enhanced the expression of BDNF and increased the level of neurogenesis in cerebral I/R animal, which might be associated with the functional recovery.

## Background

Ischemic stroke is the third leading cause of death and the first leading cause of long-term disability in the world population. Although tissue type plasminogen activator can be given during the three hours after a stroke, this treatment can only be administered to a small percentage of patients [1]. Overall, there is no effective treatment to improve functional recovery in the post-ischemic phase. The rehabilitation of patients with stroke is difficult and slow. Thus, the therapeutic strategy for post-stroke patients still remains under investigation.

It is recognized that neurogenesis play an important role in the rehabilitation after stroke in patients. The process of neurogenesis involves cell proliferation, migration, and differentiation in the ischemic region of brain [2], and is regulated by a series of signaling pathways. The transcription factor cAMP response element-binding protein (CREB) is associated with neuronal development, synaptic plasticity, and regeneration and cell survival in response to cerebral ischemia [3]. The cAMP/PKA/CREB signal transduction pathway plays a pivotal role in multiple aspects of neurogenesis [4]. Brain-derived neurotrophic factor (BDNF) regulates multiple steps of neurogenesis, including proliferation, migration, differentiation, and survival [5].

Huatuo Zaizao pill (HT), a pure natural preparation derived from plants, is a traditional Chinese medicine formulation marketed in China. HT was listed in the pharmacopoeia of the People’s Republic of China Part I (Pharmacopoeia Commission of the People’s Republic of China, 1990). The pharmacopoeia of the People’s Republic of China Part I (Pharmacopoeia Commission of the People’s Republic of China, 2015) demonstrates that HT can be used to promote the rehabilitation after stroke. Thrombosis is the common cause of ischemic stroke. With the thrombosis test of both in vitro and in vivo, HT inhibited platelet aggregation and thrombosis formation. Therefore, HT had a property to improve microcirculation disturbance and prevent cerebral thrombosis [6]. Using a cerebral I/R rat model, Liu and colleagues found that HT reduced the neurological deficit score and infarct volume. HT had protective effects on neuron and glial cells in the penumbra area [7]. Previous studies also reported that HT was effective for improving neurological functions in stroke patients [8–10]. HT improved the symptoms of hemiplegia and hemianesthesia in ischemic stroke patients by reducing the platelet aggregation and whole blood viscosity [11]. It reported that HT promoted the functional recovery of cerebral ischemia patients through decreasing the levels of endothelin, thromboxane A_2_ [12]. Xia and colleagues had analyzed a total of 10 randomized controlled trials involving 942 ischemic stroke cases. The meta-analysis revealed that HT showed a superior efficacy in treating ischemic stroke [13]. Another meta-analysis also showed that HT promoted the functional recovery of ischemic stroke patients [14]. In 2012, a phase IV, double blind, placebo-controlled, randomized, multi-center study to evaluate the efficacy of HT in improving of neural function in acute ischemic stroke had been performed (Clinical Trials. gov Identifier: NCT01758536). Despite the evidence that HT is administered to promote the rehabilitation in stroke patients, the mechanisms of pharmacological action of HT are still unclear.

HT mainly consists of five Chinese herbs: *Ligusticum chuanxiong hort*, *Borneol*, *Evodia rutaecarpa (Juss.) Benth.*, *Carthamus tinctorius L.*, and *Angelica sinensis*. Over the past decades, an increasing amount of research data showed that promoting neurogenesis is a viable therapeutic method for the treatment of stroke [15, 16]. Here, we hypothesize that HT promotes the rehabilitation of stroke by elevating neurogenesis. In this study, whether HT could promotes functional recovery and neurogenesis in a rat model of cerebral I/R injury, and the potential molecular mechanisms of these responses, such as signal transduction pathway associated with BDNF, were investigated.

## Methods

### Drug

HT was provided by GuangZhou BaiYunShan QiXing Pharmaceutical Co., LTD (Guangzhou China; batch number: 13193). The dosage for adult patients is 8–16 g (crude materials) per day. The information of quality control about HT can be found in the pharmacopoeia of the People’s Republic of China Part I (Pharmacopoeia Commission of the People’s Republic of China, 2015). The suspension of HT was prepared with 0.5% sodium carboxymethyl cellulose (CMC-Na).

### Animals

Male Sprague–Dawley rats weighing 260–280 g were provided by Beijing HFK Bioscience Co., Ltd (Beijing, China) 1 week prior to the experiment (Experimental animal certificate number: SCXK [Beijing] 2009–0015). Rats were maintained on a 12-h light/dark cycle with free access to food and water. Animal treatment and maintenance were carried out in accordance with Principles of Laboratory Animal Care (NIH Publication No. 85–23, revised 1985) and according to the rules and ethics set forth by the Ethical Committee of Yantai University. Approval for this study was granted with the registration number: 16/ECYU-13 (dated: March 16, 2013).

### Cerebral I/R model

Cerebral I/R model was carried out according to a previous method [17]. Briefly, rats were anesthetized with chloral hydrate (300 mg/kg, intraperitoneal injection) and placed in a supine position on a heated pad, with body temperature maintained at 37 °C using a rectal thermometer. A fish wire (Φ0.18 mm; Maitelifishing tackle Co., Ltd, Zhejiang, China) rounded by heating it near a flame (top diameter of 0.3 mm) was then introduced into the internal carotid artery through a small incision, and positioned approximately 18 mm from the carotid bifurcation. The fish wire was withdrawn for reperfusion 90 min later. Sham-operated group rats received sham surgery in which an identical procedure was followed but without the insertion of the fish wire.

### Neurological assessment

A neurological examination was performed according to Longa’s methods 24 h after I/R [18]. The criteria were as follows: 0, no deficit; 1, failure to extend the left forepaw fully; 2, circling to the left; 3, falling to the left; 4, no spontaneous walking with a depressed level of consciousness. The rats with scores of 0 or 4 were excluded from the study.

### Grouping and treatment

According to Longa’s score, rats were randomly assigned to five experimental groups with 12 animals in each group: sham-operated group, model group, HT at dose of 0.5 g/kg group, HT at dose of 1.0 g/kg group, and HT at dose of 2.0 g/kg group. The selected dosages of HT were based on our pilot study (Data not shown). Twenty-four hours after I/R, rats in HT groups were orally administered their respective dose of HT, once daily for 7 days. The rats in the sham and model groups were given 0.5% CMC-Na.

### Behavioral testing

Animals were handled to reduce stress before behavioral testing. All equipment, as well as the testing surface, was cleaned with 75% ethanol and left to dry before each animal was tested. The assigned groups of the animals were blinded to the investigator conducting and scoring the behavioral tests. All tests were performed in a quiet and dimly light room 7 days after HT treatment. Each test was repeated twice and separated by at least 30 min. The cylinder test assessed forelimb use asymmetry, and was performed as previously described with minor modifications [19]. Rats were placed inside a transparent glass cylinder (20 cm in diameter and 30 cm in height; RWD Life Science Co., Ltd., Shenzhen, China). A total of 20 movements were recorded during the test. Forelimb asymmetry was then calculated using the formula: 100 × (ipsilateral forelimb use + 1/2 both)/total forelimb use. A beam-walking test was applied to assess coordination and integration of motor movements as a measure of functional recovery in I/R-injured rats [20]. Rats were trained for 2 days before I/R injury. The apparatus consisted of a wooden beam (2.5 × 120 cm, positioned at a height of 50 cm by two supporting legs). A wooden box (30 cm × 30 cm × 30 cm) was set at another side to motivate the animal to cross the beam. The time taken to traverse the beam was recorded by a stopwatch. The cut-off time was taken as 60 s. The adhesive test was performed to assess the sensorimotor deficit, as previously described [21]. Adhesive tape (6 mm in diameter) was placed on the palmar surface of the paw with equal pressure, and the rat was observed in a cage. The time of the first contact of adhesive tape was measured and recorded for each forelimb. The rats were given a maximum of 2 min to perform this task.

### Immunofluorescence assay

The double labeling of 5-ethynyl-2-deoxyuridine (EdU) and neuronal nuclear protein (NeuN) was performed. EdU incorporation was detected using the Click-iT EdU Alexa Fluor® Imaging Kit (Invitrogen Life Technologies, Oregon, USA). Three rats in each group were injected intraperitoneally with EdU (50 mg/kg; diluted in 0.9% saline). Twenty-four hours later, serial coronal sections from bregma −1.80 to −4.30 were selected for immunofluorescent staining. Sections were incubated with the primary polyclonal goat against NeuN antibody (1:500, Santa Cruz Biotechnology, Santa Cruz, CA) for 30 min at 37 °C. Sections were then incubated with the secondary anti-rabbit IgG, F(ab’) 2 Fragment (Alexa Fluor® 594 Conjugate) (Cell Signaling Technology, MA, USA) for 1 h at 37 °C. Brain sections were investigated using a fluorescent microscope (Olympus IX83, Tokyo, Japan). Images were acquired with an Image-Pro Plus 6.0 software. The number of positive for EdU/NeuN within the ipsilateral peri-infract cortex were counted using a microscope (20 × objective). In all of the slices, five fields of peri-infarct regions of cortex per brain sample were quantified by an observer blinded to the experimental treatment.

### Real-time PCR (RT-PCR)

The peri-infarct regions of cortex of four rats in each group were collected on day 3 and day 7 of HT treatment. Total RNA was extracted from tissues by Trizol Reagent (Invitrogen Life Technologies). Total RNA was reversely transcribed to cDNA using the AMV First Strand cDNA Synthesis Kit (Sangon Biotech, Shanghai, China). PCR amplification was performed in 20 μL containing 1 μL of each primer and 10 μL SybrGreen RT-PCR Master Mix (Toyobo, Osaka, Japan). The amplification procedure was performed at 95 °C for 3 min followed by 40 cycles of amplification reactions at 95 °C for 15 s and 60 °C for 40 s. The primers used included BDNF (185 bp): forward, 5′ TGGGGTTAGGAGAAGTCAAGC 3′, reverse, 5′ TGTTTCACCCTTTCCACTCCT 3′; β-actin (163 bp): forward, 5′ CGTAAAGACCTCTATGCCAACA 3′, reverse, 5′ AGCCACCAATCCACACAGAG 3′. β-Actin cDNA was used as a control. Relative expression between a given sample and a reference sample was calculated using the 2^–ΔΔCt^ method [22].

### Western blotting

The peri-infarct regions of cortex were lysed in RIPA lysis buffer (50 mM Tris, 150 mM NaCl, 1% Triton X-100, 1% sodium deoxycholate, 0.1% SDS) and centrifuged at 12,000 *g* for 20 min at 4 °C. Proteins were then loaded (80 μg per lane) and subjected to 10% sodium dodecyl sulfate-polyacrylamide gel electrophoresis. The membranes were incubated overnight at 4 °C with the primary antibodies: rabbit anti-PKA (1:1000; Cell Signaling Technology, MA, USA), rabbit anti-phospho-CREB (Ser133) (1:1000; Cell Signaling Technology, MA, USA), rabbit anti-CREB (1:1000; Cell Signaling Technology, MA, USA), or rabbit anti-BDNF (1:500, Santa Cruz Biotechnology, Dallas, TX, USA). The membranes were processed with the respective horseradish peroxidase-labeled secondary antibody (1:2000; Beyotime Institute of Biotechnology). Bands were visualized using the ECL kit (Beyotime Institute of Biotechnology), and bands were quantified using Image Quant LAS 4000 (GE Healthcare Bio-Sciences AB, Tokyo, Japan). β-Actin (1:1000; Beyotime Institute of Biotechnology) served as the loading control.

### Statistical analysis

Data are presented as the mean ± SD and were analyzed by one-way analysis of variance followed by the least significant difference test. Values of *p* < 0.05 were considered statistically significant. The SPSS 17.0 Statistical Software was used for the analyses.

## Results

### Effect of Huatuo Zaizao pill on functional recovery after cerebral I/R in rats

**Fig. 1:**
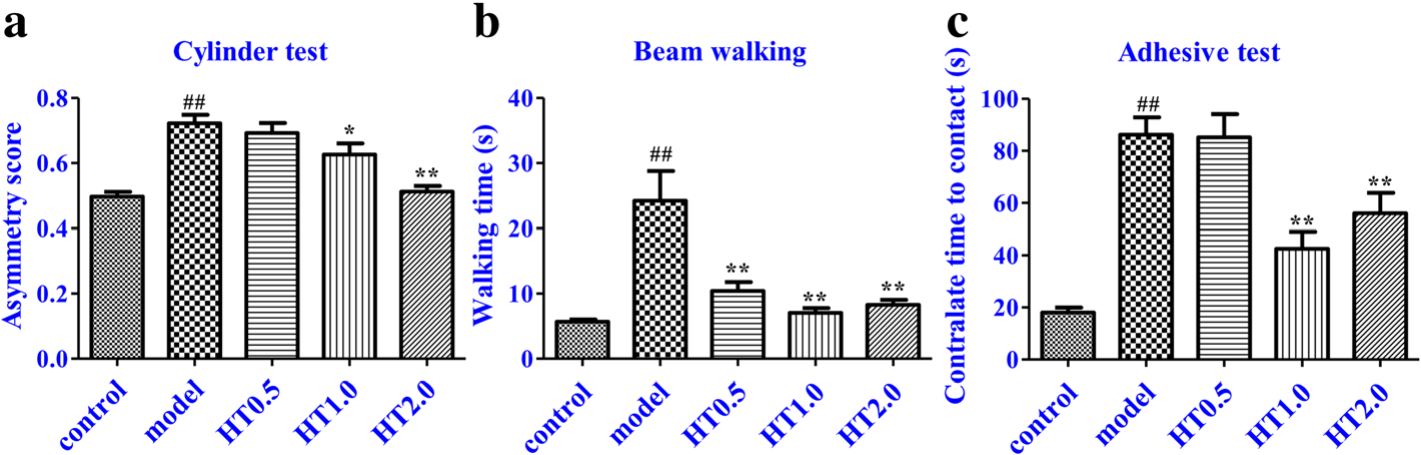
Effect of Huatuo Zaizao pill on functional recovery after 7-day cerebral I/R in rats. **a** Cylinder test. **b** Beam-walking test. **c** Adhesive test. ^##^ *p* < 0.01 vs. sham group; ^*^ *p* < 0.05, ^**^ *p* < 0.01 vs. model group

In the cylinder test, animals in the sham group did not show any paw preference during rearing. I/R injury led to a motor deficit in the impaired forepaw, which was indicated by a significant preference for the use of the unimpaired forepaw compared with sham group (*p* < 0.01). HT administration significantly improved the performance of I/R rats in the cylinder test (*p* < 0.05 or *p* < 0.01) (Fig. 1a). Animals treated with HT for 7 days spent a markedly longer time crossing the beam compared with sham group (*p* < 0.01). The walking time in the HT groups was significantly shorter than that of the model group without HT treatment (*p* < 0.01) (Fig. 1b). The functional recovery of sensorimotor impairments was assessed as the time needed to contact the adhesive tape by the impaired forepaw. Rats in the model group significantly prolonged the contact time compared with the sham group (*p* < 0.01). I/R rats with HT treatment had a significantly improved performance (*p* < 0.01) (Fig. 1c).

### Effect of Huatuo Zaizao pill on neurogenesis after cerebral I/R in rats

**Fig. 2:**
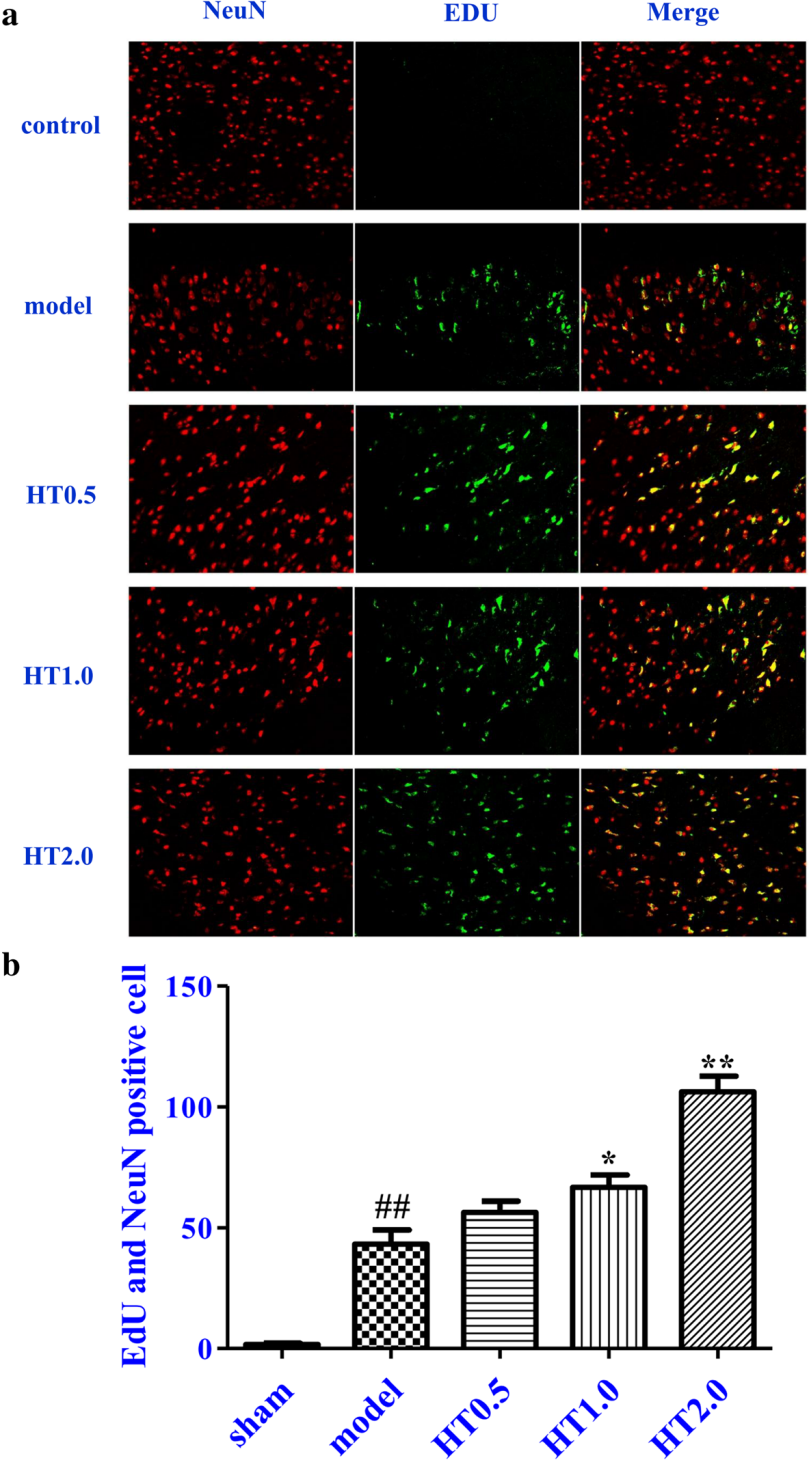
Effect of Huatuo Zaizao pill on neurogenesis after 7-day cerebral I/R in rats. **a** EdU-positive cells (green) with NeuN-positive cells (red). **b** Quantification of the number of cells with co-localized EdU and NeuN. ^##^ *p* < 0.01 vs. sham group; ^*^ *p* < 0.05, ^**^ *p* < 0.01 vs. model group

The co-localization of EdU and NeuN was examined after 7-day treatment in the peri-infarct regions of cortex of rats. Few co-stained cells were observed in the sham group. A higher number of co-stained cells were found in the model group compared with the sham group (*p* < 0.01). HT treatment significantly increased the number of cells co-labeled with EdU and NeuN compared with the model group (*p* < 0.05 or *p* < 0.01) (Fig. 2).

### Effect of Huatuo Zaizao pill on the level of BDNF mRNA in I/R rats

**Fig. 3:**
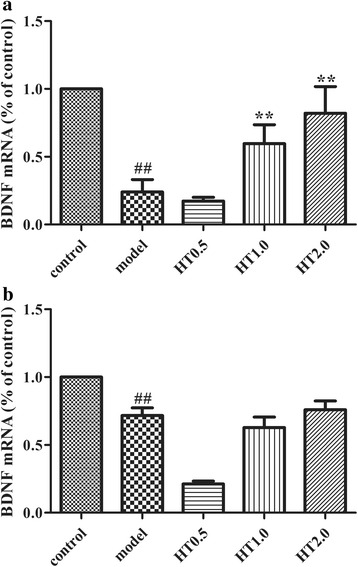
Effect of Huatuo Zaizao pill on the level of BDNF mRNA in the peri-infarct regions of cortex in cerebral I/R rats. **a** The level of BDNF mRNA on day 3 of HT treatment. **b** The level of BDNF mRNA on day 7 of HT treatment. ^##^ *p* < 0.01 vs. sham group; ^**^ *p* < 0.01 vs. model group

The level of BDNF mRNA in peri-infarct regions of cortex was detected on day 3 and day 7 treatment. Compared with model rats, the mRNA expression of BDNF was significantly augmented in the peri-infarct regions of cortex of rats treated with 1.0 or 2.0 g/kg HT for 3 days (*p* < 0.01). However, a 7-day HT treatment did not significantly alter BDNF mRNA expression compared with the model group (Fig. 3).

### Effect of Huatuo Zaizao pill on the PKA, p-CREB/CREB, and BDNF expressions in I/R rats

**Fig. 4:**
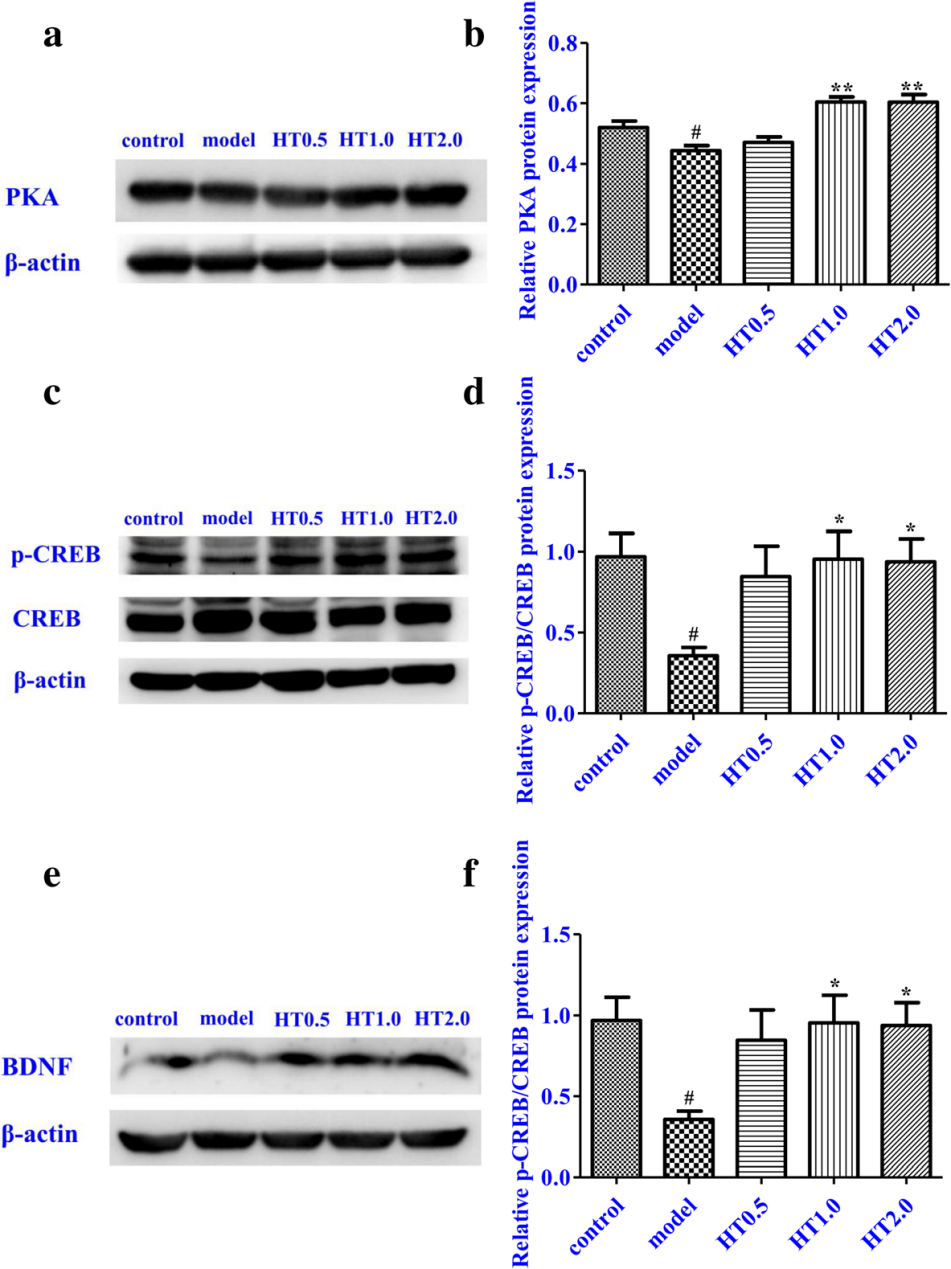
Effect of Huatuo Zaizao pill on the protein expression of PKA, p-CREB/CREB, and BDNF in peri-infarct regions of cortex in cerebral I/R rats. **a**, **c**, and **e** representative photographs of PKA, CREB, p-CREB, and BDNF. **b**, **d**, and **f** quantification of protein bands for PKA, CREB, p-CREB, and BDNF. Three rats per group. ^#^ *p* < 0.05 vs. sham group, ^*^ *p* < 0.05; ^**^ *p* < 0.01 vs. model group

The protein expressions of PKA, CREB, p-CREB, and BDNF in peri-infarct region of cortex were examined on day 7 of HT treatment. Western blotting showed that I/R injury markedly reduced the level of expression of PKA compared with the sham group (*p* < 0.05). Compared with model group, HT treatment significantly increased PKA expression (*p* < 0.01). I/R injury reduced p-CREB compared with the sham group, and HT significantly promoted p-CREB compared with the model group (*p* < 0.05). I/R injury significantly reduced BDNF expression compared with sham group (*p* < 0.05), and HT treatment markedly increased BDNF compared with model group (*p* < 0.01) (Fig. 4).

## Discussion

In this study, the effects of HT on functional recovery in I/R rats were investigated. Based on previous methodologies for evaluating neurological function after brain injury [23], the behavioral performance of HT in ischemic rats was assessed by the cylinder test, beam-walking test, and adhesive test. The results showed that a 7-day treatment with HT attenuated the preference for the use of the unimpaired forepaw in the cylinder test, reduced the walking time in the beam-walking test, and decreased the contact time in adhesive test. These findings demonstrated that HT could promote functional recovery after cerebral I/R in rats.

Neurogenesis plays a key role in promoting functional recovery after stroke [24]. Whether HT could promote functional recovery by increasing neurogenesis in a rat model of cerebral I/R injury is still unclear. The effects of HT on neurogenesis were investigated via double immunofluorescence staining. EdU incorporating into cellular DNA was used to detect DNA synthesis during the S phase of the cell cycle [25]. NeuN is a marker of mature neurons. EdU labeled cells co-expressed NeuN suggesting that newborn cells differentiate into neurons after genesis [26]. The results of double staining of EdU and NeuN showed a significant increase in the number of newborn neurons in HT-treated animals. This result indicates that HT may increase the neurogenesis, thereby to promote functional recovery after stroke.

CREB is a transcriptional factor associated with neurological disease-related genes [27]. It is abundant in the central nervous system and particularly in neurons. CREB phosphorylation is implicated in both neuronal plasticity and neurotrophin-mediated neuronal survival [28]. PKA phosphorylates CREB and phosphorylated CREB may then bind to the CRE element of the promoter and consequently, promote the transcription of BDNF [29]. Tanaka et al. have shown a rapid and transient increase in CREB phosphorylation within the ischemic core territory following cerebral ischemia. Furthermore, p-CREB is decreased significantly following 12 and 24 h of cerebral I/R [30]. Heightened expression of BDNF plays an important role in modulating neurogenesis after stroke [31]. There is a possible connection between the PKA/CREB signal transduction pathway and BDNF [32]. In this study, the effects of HT on PKA/CREB and BDNF expression were explored in the peri-infarct regions of cortex of rats. The results showed that protein expression of PKA was significantly decreased after cerebral I/R injury. HT treatment reversed this effect. Furthermore, in the peri-infarct regions of cortex, the total protein level of CREB did not alter, but p-CREB was found to be significantly reduced in the model group. HT treatment could increase CREB phosphorylation, thus p-CREB/CREB was raised.

Gene expression levels can vary in different brain areas after stroke and across the different stages of the ischemic cascade. Previous report showed there was a transient up-regulation of BDNF mRNA during the 24 h after the ischemic insult [33]. It was also reported that an increase in BDNF mRNA expression occurs between 1 and 3 days, returning to basal level 4 days after cerebral ischemia [34]. However, this study found that BDNF was down-regulated in ischemic brain tissue at 4 day after cerebral I/R. We supposed the disparity may be due to the difference of ischemic time and sampling area. In consistent with Lee and colleagues, we found that BDNF mRNA decreased significantly in the ischemic brain tissues at 7 d after cerebral I/R, and that BDNF protein showed a similar expression pattern to its mRNA [35]. Our results showed that the peri-infarct regions of cortex in 3-day HT-treated rats revealed a higher BDNF mRNA. No significant alterations in BDNF mRNA were found between the HT group and the model group at this time-point, while a 7-day treatment of HT increased the protein levels of BDNF in the peri-infarct regions of cortex. Protein production and maintenance requires a series of processes. Therefore, proteins may not occur in proportion to their relative mRNA levels [36]. Based on previous reports and our current findings, it is reasonable to suppose that HT treatment increased the mRNA then protein level of BDNF through the activation of the cAMP/PKA/CREB signal pathway.

Neurogenesis and angiogenesis after stroke are linked together and coordinated. For instance, neuroblasts migrate from the SVZ region to the ischemic area where post-stroke angiogenesis occurs, and these neuroblasts migrate closely with cerebral vessels. Many migrating neuroblasts are found to localize specifically to blood vessels in areas of active vascular sprouting and remodeling in the infarct boundary [37]. It is also reported that neurogenesis and angiogenesis are coupled to enhance central nervous repair after ischemic stroke [38]. Although our data show that HT promotes neurogenesis in stroke rats, further studies are warranted to determine the effects of HT on angiogenesis.

## Conclusion

HT is a traditional Chinese medicine used for management of stroke. This study distinctly demonstrated that HT promoted behavioral recovery of rats with I/R induced cerebral damage. Furthermore, HT enhanced the expression of BDNF and increased the level of neurogenesis in cerebral I/R animal. These findings may elucidate the pharmacological basis of the action of HT that help rehabilitate patients with stroke.

## Abbreviations

BDNF: Brain-derived neurotrophic factor
BSA: Bovine serum albumin
CMC-Na: Sodium carboxymethyl cellulose
CREB: cAMP response element-binding protein
p-CREB: Phosphorylated cAMP response element-binding protein
DCX: Doublecortin
ECA: External carotid artery
EdU: 5-Ethynyl-2-deoxyuridine
HT: Huatuo Zaizao pill
I/R: Ischemia-reperfusion
PBS: Phosphate-buffered saline
PKA: Protein kinase A
RT-PCR: Real-time polymerase chain reaction
SVZ: Subventricular zone
